# Usage of Fermental Traps for the Study of the Species Diversity of Coleoptera

**DOI:** 10.3390/insects12050407

**Published:** 2021-04-30

**Authors:** Alexander B. Ruchin, Leonid V. Egorov, Anatoliy A. Khapugin

**Affiliations:** 1 Joint Directorate of the Mordovia State Nature Reserve and National Park ”Smolny”, 430005 Saransk, Russia; platyscelis@mail.ru (L.V.E.); hapugin88@yandex.ru (A.A.K.); 2 Prisursky State Nature Reserve, 428034 Cheboksary, Russia; 3 Institute of Environmental and Agricultural Biology (X-BIO), Tyumen State University, 625003 Tyumen, Russia

**Keywords:** fermental traps, beer traps, Coleoptera, fauna, biodiversity, occurrence

## Abstract

**Simple Summary:**

This study describes how simple traps can be used to study tree crowns and undergrowth at low altitudes. They are used with bait made of fermenting liquids (beer, wine) with the addition of sugar and other carbohydrates. The research was conducted between 2018 and 2020 in several regions of Russia. It was possible to identify 294 species from 45 Coleoptera families during this time. Simple traps have been shown to be highly effective, and can be used to study insect biodiversity in forest ecosystems.

**Abstract:**

The possibilities of applying various methods to study Coleoptera give unexpected and original results. The studies were carried out with the help of fermental crown traps in 2018–2020 on the territory of eight regions in the central part of European Russia. The biodiversity of the Coleoptera that fall into crown traps includes 294 species from 45 families. The number of species attracted to the fermenting bait is about a third of the total number of species in the traps (this is 97.4% of the number of all of the caught specimens). The largest number of species that have been found in the traps belong to the families Cerambycidae, Elateridae and Curculionidae. The most actively attracted species mainly belong to the families Cerambycidae, Nitidulidae and Scarabaeidae. The species of these families are equally attracted by baits made of beer, white and red wines. In order to identify the Coleoptera biodiversity of a particular biotope, two-year studies are sufficient, and they should be carried out throughout the vegetation season. Especially good results can be obtained from studies of rare species that are actively attracted by such baits. It is possible to study the vertical–horizontal distribution of Coleoptera fauna in individual biotopes.

## 1. Introduction

Forests are biologically diverse ecosystems that represent some of the richest communities of living organisms on Earth. Due to the diversity of these ecosystems, they are home to a significant species diversity of insects [1,2,3,4,5,6,7,8]. While many insect species thrive, some forest species are on the verge of extinction due to forest degradation, pollution, fragmentation, changes in tree composition, climate change, and other factors, such as fires, tree felling, and draining [9,10,11,12,13,14,15,16]. The species diversity of Coleoptera forest ecosystems is very large, and knowledge about this biodiversity is constantly being updated through the use of a wide variety of studying methods [17,18,19,20,21,22].

Entomological net mowing, window traps, pitfall traps, light traps, and Malaise traps are key methods for studying Coleoptera biodiversity in forest systems [23,24,25,26,27,28]. Most of these methods are easy to use, and therefore a huge number of studies are based on these research methods. At the same time, these methods are usually used at the level of human growth. These methods are quite accessible, and are actively used to study insects of the soil and herbaceous tiers, as well as—to a certain extent—shrubs and undergrowth. In open ecosystems, such as grasslands, steppes, and deserts, these methods are sufficient to study biodiversity [29,30,31,32].

However, these methods do not always accurately assess the biodiversity of Coleoptera in individual forest areas or in specific forest tiers. This is especially true for the study of the upper tiers of the forest, which are often inaccessible to the entomologist with a net. Some collection methods are quite labor-intensive, and are therefore rarely used by entomologists. Fermental crown traps with various baits are an additional and well-established method of studying the biodiversity of Coleoptera in the forest canopy [33]. Bait trapping for insects is discussed in many general entomological texts, and ranges from techniques such as ‘sugaring’ and pheromone traps to using ‘natural’ organic baits such as carrion and dung [34]. Traps with baits made of fermenting liquids, such as wine, molasses, and beer, with the addition of bananas, apples, sugar and other natural fillers have proven effective in detecting many Coleoptera families [35,36,37,38,39,40,41,42,43]. Previously, a mixture of beer with sugar, honey and jam was successfully used as bait [44,45,46]. Using such original and unusual collection methods, it is possible to find new species not only for the region, but also for science [47,48]. This study presents the results of studying Coleoptera using fermental crown traps in various regions of Central Russia and the Volga region.

## 2. Materials and Methods

### 2.1. Placement of Traps

The traps are a plastic 5 L container with a window cut out of it on one side at a distance of 10 cm from the bottom. With the help of a load, a rope with a tied trap was thrown onto a tree branch at a height of 5 to 12 m from the soil surface [46,49]. As bait, fermenting beer, white and red dry wine were used, with an addition in the form of honey, jam or sugar.

The traps were placed in eight regions: the Republic of Mordovia, and the Tambov, Saratov, Ryazan, Vladimir, Nizhny Novgorod, Ulyanovsk, and Penza regions in 2018 (from June to August), in 2019 (from April to October), and in 2020 (from April to October). The volume of material for this article is presented in Table 1.

### 2.2. Usage of Attractive Liquids

Over several series of experiments, the most attractive liquids for Coleoptera were determined. The attractive liquids were white wine, red wine, and beer. The attraction mixture consisted of these liquids, with or without added sugar. The following variants of mixtures were studied:

(1) beer with sugar (BS),
(2) beer without sugar (B),
(3) red wine with sugar (RvS),
(4) red wine without sugar (Rv),
(5) white wine with sugar (WvS),
(6) white wine without sugar (Wv).

These experiments were carried out from April to August (they were repeated 10 times). All of the traps in each series of experiments were located on oak trees at the same height (5.5–6 m) at a close distance from each other (no more than 10–15 m). Each repetition of the experiment (exposure) was carried out for 7–10 days. Each repetition was carried out within one biotope (on an area of no more than 500 m^2^).

### 2.3. Calculations and Used Terms

Several terms were used to determine the effectiveness of traps. Occurrence: the ratio of the number of samples in which a species (taxonomic group) is present to the total number of samples (expressed in %). Exposure time: the period between hanging a trap and taking samples for analysis (expressed in days). Bait: a liquid that attracts insects, located in a trap, consisting of various mixtures (beer, wine, water) and natural fillers such as solid and liquid food additives (sugar, honey, jam).

### 2.4. Format

The classification of the family-group taxa used in this checklist follows predominantly Bouchard et al. [50], with subsequent additions [51]. Changes from the Catalog of Palaearctic Coleoptera are taken into account [52,53,54,55,56,57,58], as well as data on the Cucujoidea from the article by Robertson et al. [59], and Curculionoidea from the publication of Alonso-Zarazaga et al. [60]. In order to clarify the nomenclature, the cited articles were used, as well as the Catalog of Palaearctic Coleoptera [61,62]. Years of description of some species are specified by Bousquet [63]. All of the species identification was carried out by L.V. Egorov.

## 3. Results

**Figure 1:**
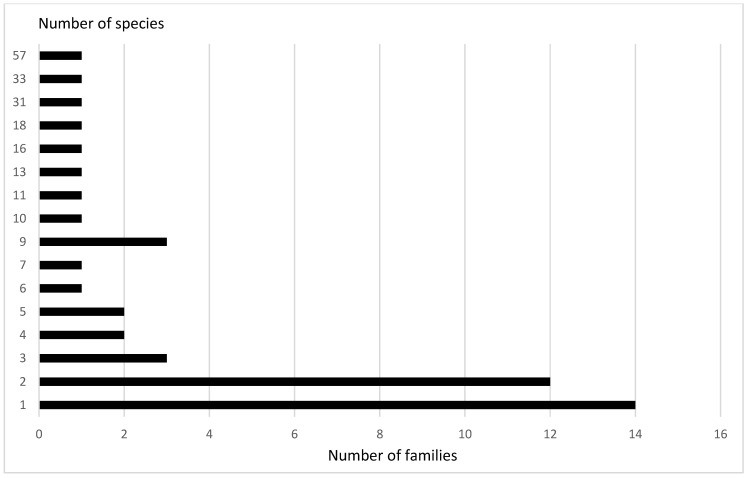
Distribution of the families by the number of captured species in the beer traps.

During the experiments in 2018–2020, more than 33,000 Coleoptera (Appendix A) specimens fell into our traps. In total, 294 species from 45 families were recorded in the traps (Figure 1). About 1500 specimens could not be identified with regard to the species (mainly from the families Staphylinidae and Nitidulidae).

The largest number of species that were found in the traps belonged to the families Cerambycidae (57 species), Elateridae (33 species) and Curculionidae (31 species). However, the overwhelming number of families were represented in our catches by single species: only one species was recorded among 14 families (Staphylinidae, Hydrochidae, Monotomidae, Cucujidae, Lycidae, Brentidae, Attelabidae, Aderidae, Laemophloeidae, Boridae, Lymexylidae, Silvanidae, Mordellidae, Salpingidae), two species were recorded among 10 families (Erotylidae, Throscidae, Mycetophagidae, Scraptiidae, Pyrochroidae, Anthribidae, Cerylonidae, Melandryidae, Dytiscidae, Eucnemidae), and three species were recorded among three families (Scirtidae, Latridiidae, Ptinidae).

Based on our long-term research, we can distinguish between species that are attracted by the mix, and random species that fall for some other random reasons (for example, they stumble on the transparent walls of the trap or fly to the water). In some cases, when installing a trap, and during its prolonged exposure, especially in sunny places, the processes of the rotting of trapped insects can occur. This leads to the trapping of species that are attracted by carrion. We distinguish this group separately.

**Figure 2:**
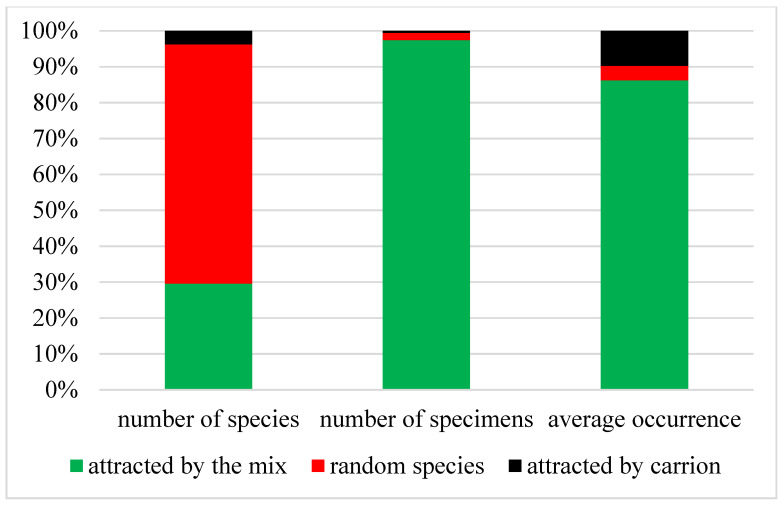
The ratio of the number of species, the number of specimens and the average occurrence of species, depending on the ability to attract to the bait.

Thus, we conditionally distinguished three groups of Coleoptera species that fall into traps (Figure 2). The number of species attracted by the mix was 29.6% of the total number of species in the traps. However, they accounted for 97.4% of the number of samples that were identified. The average occurrence of these species exceeded the occurrence of random species by 21 times. The high occurrence of species that are attracted by carrion was noted. They probably react quickly enough to the prey and fall into the traps.

**Figure 3:**
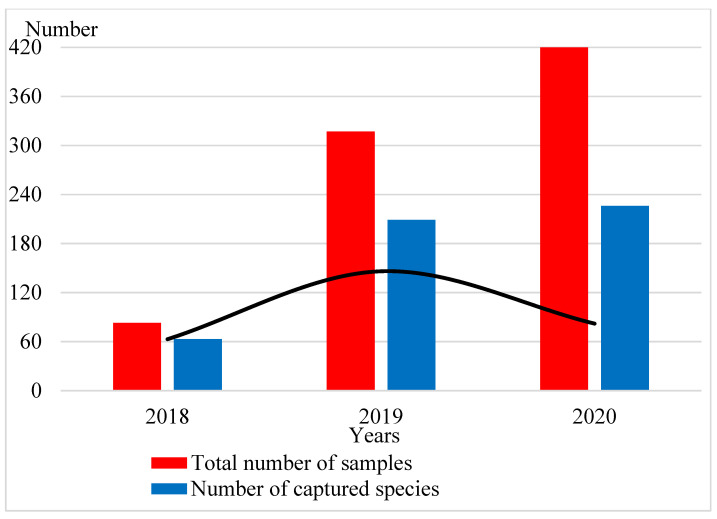
Dependence of the number of captured species on the number of traps by year.

As studies have shown (Figure 3), the increase in the number of traps in the third year did not have the same effect as in the first two years. From 2018 to 2020, we increased the number of traps set for the study of Coleoptera. We also increased the number of regions where these traps were located. It turned out that the number of species that fall into the traps increased significantly in the second year of the study, with an increase in the number of traps. However, in the third year of research, despite the higher number of traps, the number of new species that had not been caught before decreased. New species were trapped in 2020 due to an increase in the number of regions. Thus, in the third year, the number of new species caught in the traps decreased. They already included random and/or very rare species that live in this biotope. It can be concluded that two-year studies will be sufficient to study the biodiversity of a particular biotope or a small region. We used several compositions of mixtures, in which the basis was red wine, white wine or beer. Sugar and yeast were added as additives to this bait.

**Figure 4:**
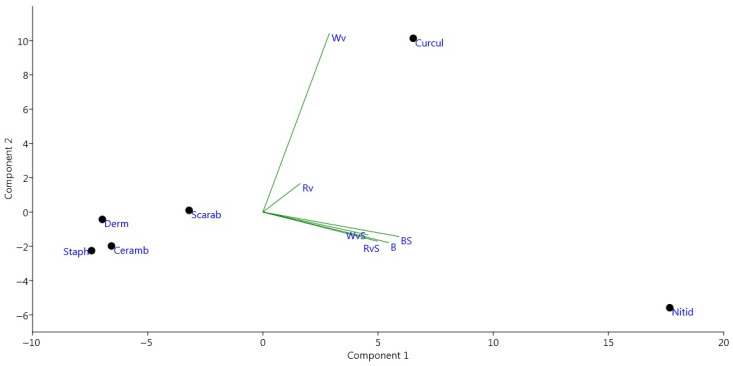
Canonical analysis of the number of registered specimens from different families, depending on the bait composition (beer with sugar (BS), beer without sugar (B), red wine with sugar (RVs), red wine without sugar (Rv), white wine with sugar (WvS), white wine without sugar (Wv)). Families: Derm—Dermestidae, Scarab—Scarabaeidae, Staph—Staphylinidae, Nitid—Nitidulidae, Ceramb—Cerambycidae, Curcul—Curculionidae.

Figure 4 shows the same direction of the effects of factors with some variance from Wv (above) to B (below all) for families and for species. It turned out that the number of Curculionidae specimens (mainly due to *Anisandrus dispar*) increases when catching with white wine without sugar, and to a lesser extent with red wine without sugar. At the same time, the number of Nitidulidae specimens is not related to these factors, but their catchability increases with all other factors. As for the other families, they are all equally attracted to baits from different mixtures. Thus, the Dermestidae, Scarabaeidae, Staphylinidae, and Cerambycidae are similarly attracted to beer- and wine-based baits.

**Figure 5:**
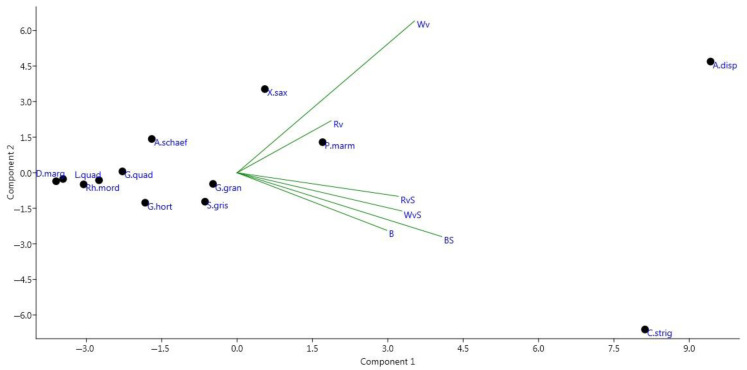
Canonical analysis of the number of recorded specimens of various species, depending on the composition of the bait (see the caption to Figure 4). Species: A.schaef—*Attagenus schaefferi* (Dermestidae), P.marm—*Protaetia marmorata* (Scarabaeidae), D.marg—*Dalopius marginatus* (Elateridae), C.strig—*Cryptarcha strigata* (Nitidulidae), G.hort—*Glischrochilus hortensis* (Nitidulidae), G.quad—*Glischrochilus quadripunctatus* (Nitidulidae), G.gran—*Glischrochilus grandis* (Nitidulidae), S.gris—*Soronia grisea* (Nitidulidae), L.thor—*Leptura thoracica* (Cerambycidae), L.quad—*Leptura quadrifasciata* (Cerambycidae), Rh.mord—*Rhagium mordax* (Cerambycidae), A.disp—*Anisandrus dispar* (Curculionidae), X.sax—*Xyleborus saxesenii* (Curculionidae).

Figure 5 shows the number of recorded specimens of various species, depending on the composition of the bait. It turned out that Cryptarcha strigata is better caught using the largest number of mixtures (B, BS, WvS, RVs), while Wv and Rv attract *Anisandrus dispar*, *Protaetia marmorata* and *Xyleborus saxesenii* to a lesser extent. However, most of the studied species were almost equally lured by the different mixtures.

Thus, the species composition of Coleoptera from fermental crown traps differs from those caught by other methods. Previously, such traps have been recommended for use in the study of rare insect species [46]. For example, we present the results of the study of rare species of Coleoptera, which are included or recommended in the Red Books of some regions [6,64,65,66,67,68,69,70,71] and the Red Data Book of Russia [72] (Table 2).

In total, 18 species of Coleoptera which are included or are planned to be included in the Red Data Books, from nine families, were indicated in the studies. Especially significant are the results for the species that actively fly into crown traps for beer bait (*Quedius dilatatus*, *Gnorimus variabilis*, *Protaetia fieberi*, *Protaetia marmorata*, *Protaetia speciosissima*, *Elater ferrugineus*, *Purpuricenus kaehleri*, *Necydalis major*, *Leptura aurulenta*, *Aromia moschata*). The number of finds of such species increases significantly with an increase in the number of traps set. The use of such baits makes it possible to clarify even the status of species that have been included in the Red Data Books, and to suggest measures for their protection.

## 4. Discussion

Bait traps are an effective tool for the study of the insect fauna of the upper tiers of forests. Forest crowns are usually studied to a lesser extent than the soil and herbal layer [73,74]. Forest canopies did not attract researchers for a long time due to the logistical difficulties of reaching the tree crowns and the subsequent sampling problems. However, there were original research methods, including slingshots, crossbows, ropes, ladders, and networks of cranes, towers and passages which facilitate the work [75,76,77,78].

The active attraction of insects by baits based on fermenting beer and wine with the addition of sugar and other sweet substances, as well as fruits, can be explained. Many insects have receptors that perceive carbohydrates. According to many modern studies, insects have an excellent ability to perceive sugar [79,80,81]. Sweet carbohydrates play a crucial role in the life of insects as valuable energy and food resources. Insects always use the perception of sugars to assess the nutritional value of their food. Sugar and its decomposition products form the primary stimulatory signal for insect nutrition [82,83]. The use of traps with our baits is based on the perception of sugars as food components. We note that many other substances (alcohols, ketones, and other volatile substances) are released during fermentation, which can also attract insects [84,85,86].

However, not all insects are equally lured into such traps. There are species that are particularly common in traps, but a large number of specimens from the total number of species is attracted to bait in fermental crown traps.

*Protaetia marmorata* (Scarabaeidae)’s average occurrence was 72.2% over three years. This species inhabits various types of forests, and is found in parks, shelterbelts, and other biotopes [87,88,89]. Its larval development occurs in the hollows of dead deciduous trees for three years [87,89]. In beer traps, this is the most common type. It actively flies into fermenting bait.

*Cryptarcha strigata* (Nitidulidae)’s average occurrence was 51.2% over three years. It inhabits deciduous and mixed forests. Its imagos are often found near the effluents of the fermenting sap of *Q. robur*, where the preimaginal phases of this species develop. Occasionally, they are found on the leaking sap of *P. tremula* [90]. In beer traps, they are often found, sometimes in a very significant number.

*Glischrochilus grandis* (Nitidulidae)’s average occurrence 33.6% was over three years. It inhabits a wide variety of forest biocenoses. It is common on the leaking sap of various trees where the larvae develop. It is also known from tinder plants and from rotten berries, and develops on various decaying substrates [91,92,93,94]. It is caught in traps with vinegar bait [95]. The peak number in beer traps is typical in May–June, and single specimens are caught during all seasons.

*Protaetia fieberi* (Scarabaeidae)’s average occurrence was 30.9% over three years. It inhabits various deciduous and mixed forests, and is common in parks and deciduous second-growth forest. The larvae of this species are supraciliary. The larvae and frass inhabit the tree hollows (*Quercus*, *Tilia*, *Fagus*, *Salix*, *Populus*) made by various species of woodpeckers, owls, and small mammals [96]. Previously, it was considered rare. However, our studies have shown that this species occurs regularly in different biotopes in the center of European Russia [97].

*Leptura quadrifasciata* (Cerambycidae)’s average occurrence was 30.7% over three years. It is found in a wide range of biotopes. The larvae develop in dead or rotting wood, especially in the lower parts of standing trees, stumps, fallen trunks and the branches of various trees (alder, aspen, poplar, birch (birch may be preferred to other trees), hazel, oak, sallow, beech, willow, and elder). It inhabits wet or dry woodlands [98].

*Soronia grisea* (Nitidulidae)’s average occurrence was 28.6% over three years. It is confined to oak forests and mixed stands with the presence of oak, where it is often found on the sap of *Q. robur* and *Salix* [90,93,94]. In Turkey, it was also caught on baits with beer in mixed forests and pine forests [99]. The peak number in beer traps is typical in May–June, and single specimens are caught during all seasons.

*Glischrochilus hortensis* (Nitidulidae)’s average occurrence was 28.5% over three years. It inhabits deciduous and mixed forests. Its imago are found on the fermenting sap of *Q. robur* and under the bark of fallen and dying trees of *B. pendula* and *P. tremula*. Larvae develop under the bark of dying and damaged trees of *B. pendula*, *P. tremula*, and *Q. robur* and in their fermented sap, and can also occur on fermented berries, vegetables, and mushrooms [90,94,100]. The peak number in beer traps is typical in May; single specimens are caught during all seasons.

*Rhagium mordax* (Cerambycidae)’s average occurrence was 26.2% over three years. It is one of the most common species. It inhabits mixed, deciduous forests, and pine forests of various types [101]. Its larvae develop under the bark of dead pine and deciduous trees [102]. It is regularly found in beer traps from the end of April to July.

*Leptura thoracica* (Cerambycidae)’s average occurrence was 25.2% over three years. It is considered a polyphage of deciduous trees (*Populus*, *Betula*, *Tilia*, *Salix*, *Fagus*). The larvae inhabit the dead, rotten wood of thick trunks [103,104,105]. It has been observed that mass collections of this species occur in places with a predominance of *Betula* sp. in the stand [106]. The species was previously found in single specimens when studying the territory by conventional methods (net fishing, light fishing, window fishing) [101]. The use of beer traps has shown that the species is quite common in a wide range of biotopes.

*Cetonia aurata* (Scarabaeidae)’s average occurrence was 18.0% over three years. It inhabits a wide range of biotopes. It is often found on the flowers from the families Umbelliferae, Rosacea, and Asteraceae, where they feed on pollen and nectar [107]. The larvae develop in rotting wood and decaying plant substances [108]. It is found in beer traps which are placed at low altitudes, most often up to 5 m. It is rarely caught in very high-placed traps.

*Quedius dilatatus* (Staphylinidae)’s average occurrence was 15.4% over three years. This species is associated with *Vespa crabro* nests, where its larvae feed on Diptera larvae in the nest debris [109]. Therefore, it is often found on tree trunks, near the nests of Vespa crabro, but it is also often observed in other places. It inhabits forest biocenoses. It is often observed on the trunks of trees in the leaking sap [110]. In beer traps, it is caught in summer.

*Protaetia cuprea volhyniensis* (Scarabaeidae)’s average occurrence was 14.9% over three years. It inhabits a wide variety of forest biocenoses. This is a myrmecophilic species; the larvae usually develop in active and abandoned anthills, and sometimes in sawdust and garbage heaps. It is quite common on flowering plants [111,112].

All of these species were trapped annually with approximately the same occurrence. It is highly likely that they will be caught if traps with fermenting liquid are set in a certain biotopes during the season of activity of these species. On the other hand, those species can be caught that are very rare in the studied territory. For example, very rare species (*Allonyx quadrimaculatus*, *Anoplodera rufipes ventralis*, *Leptura aurulenta*, *Purpuricenus globulicollis*) are practically not caught, despite the use of different methods. Other species which are not often detected with the help of other methods (*Quedius dilatatus*, *Protaetia affinis*, *Protaetia fieberi*, *Protaetia speciosissima*, *Elater ferrugineus*, *Ctesias serra*, *Globicornis emarginata*, *Nacerdes carniolica*, *Purpuricenus kaehleri*, *Aromia moschata*, *Leptura thoracica*, *Necydalis major*, *Xylotrechus pantherinus*) are well lured by wandering baits, and with the help of these baits their numbers can be estimated.

The quality of the bait can affect both the number of individuals caught and the species. Many effective traps have been suggested in several studies. For example, pineapple traps attract *Scyphophorus acupunctatus* (Curculionidae) better than fermented maguey [113]. The vinegar-ethanol-apple mixture was much more effective in attracting *Eucryptorrhynchus scrobiculatus* (Curculionidae) [114]. Bardiani et al. [115] successfully used baits made from various wines, beer, and banana puree to catch *Lucanus cervus* (Lucanidae). Traps with baits made of beer, palm wine, and various fruits (banana, mango, papaya, or pineapple) were successfully used to catch Cetoniinae (Scarabaeidae) [116]. Other studies [117] have shown that there was the greatest species richness and abundance of Cetoniinae in traps with bait made from banana juice and sugar cane, pineapple and sugar cane, and only sugar cane juice compared to other baits. These results showed the importance of sugar cane juice, used either in isolation or as an additive in the fruit fermentation process, for effective sampling [117]. A mixture of banana, brown sugar, molasses, and baker’s yeast was used to study Cerambycidae fauna [118]. On the other hand, Allemand and Aberlenc [35] used a mixture of beer and red wine in equal amounts to capture beetles without adding other ingredients (fruit flavors, sugar, honey). Thus, the species composition of Coleoptera in bait traps clearly depends on the specific composition of the bait itself [119]. Different fishing methods, when used correctly, can be effective tools, for example, in monitoring biodiversity and studying rare insect species that are difficult to detect by other methods [44,120,121].

## 5. Conclusions

The biodiversity of Coleoptera that fall into crown traps is large. Over a three-year period, we observed 294 species from 45 families. Most families are represented by between one and three species. The number of species actively attracted to the bait is about a third of the total number of species in the traps. At the same time, they account for 97.4% of the number of specimens. Two-year studies are sufficient to identify the Coleoptera biodiversity of a particular biotope. However, they need to be conducted during the entire insect activity season. Such studies will fully characterize the Coleoptera fauna. The largest number of species found in the traps belonged to the families Cerambycidae, Elateridae, and Curculionidae. However, the actively attracted species mainly belonged to the families Cerambycidae, Nitidulidae and Scarabaeidae. The species of these families are equally attracted by baits made of beer, or white and red wines.

We recommend the use of fermental crown traps with beer and wine for ecological studies of the Coleoptera fauna. This method can be applied to study the seasonal and spatial characteristics of the fauna. Especially good results can be obtained from studies of rare species that are actively attracted by such baits. It is possible to study the vertical–horizontal distribution of fauna in individual biotopes.

## Figures and Tables

**Table 1:** The quantity of the collected material in the regions of Russia *.

Region	2018	2019	2020	Total
Republic of Mordovia	83 1750	266 10,617	226 10,901	**575** **23,268**
Penza region	0 0	18 495	86 4968	**104** **5463**
Ulyanovsk region	0 0	20 278	32 1767	**52** **2045**
Nizhny Novgorod region	0 0	13 265	29 880	**42** **1145**
Vladimir region	0 0	0 0	21 143	**21** **143**
Ryazan region	0 0	0 0	19 850	**19** **850**
Saratov region	0 0	0 0	4 60	**4** **60**
Tambov region	0 0	0 0	3 295	**3** **295**
Total	**83** **1750**	**317** **11,655**	**420** **19,864**	**820** **33,269**

* the number of traps installed is above the line; the number of recorded beetle samples is below the line.

**Table 2:** Occurrence of rare species (numbers indicate the number of rare species found in the region according to the fermental crown trap records).

Species	Red Data Book of Russia	Red Data Book							
		Vladimir Region	Ryazan Region	Republic of Mordovia	Penza Region	Nizhny Novgorod Region	Ulyanovsk Region	Saratov Region	Tambov Region
**Carabidae**									
*Lebia marginata* (Geoffroy, 1785)	–	–	–	1 (2) *	–	–	–	–	–
**Staphylinidae**									
*Quedius dilatatus* (Fabricius, 1787)	–	–	–	57 (10)	–	–	10 (5)	–	–
**Silphidae**									
*Dendroxena quadrimaculata* (Scopoli, 1771)	–	0 (5)	–	10 (3)	–	–	–	–	–
**Lucanidae**									
*Lucanus cervus* (Linnaeus, 1758)	+	0 (1)	–	0 (3)	0 (25)	0 (2)	3 (43)	0 (30)	0 (7)
**Scarabaeidae**									
*Gnorimus variabilis* (Linnaeus, 1758)	–	0 (6)	0 (2)	29 (6)	2 (6)	–	0 (9)	0 (10)	0 (2)
*Osmoderma barnabita* Motschulsky, 1845	+	0 (3)	0 (9)	3 (4)	0 (7)	0 (11)	0 (7)	0 (5)	0 (1)
*Protaetia fieberi* (Kraatz, 1880)	+	7 (4)	5 (–)	125 (12)	48 (6)	11 (–)	40 (–)	2 (9)	1 (–)
*Protaetia marmorata* (Fabricus, 1792)	–	–	15 (7)	–	–	–	–	–	–
*Protaetia speciosissima* (Scopoli, 1786)	+	2 (–)	1 (2)	25 (8)	20 (11)	4 (1)	9 (25)	1 (14)	1 (4)
**Elateridae**									
*Elater ferrugineus* Linnaeus, 1758	+	–	–	6 (2)	–	–	1 (–)	0 (1)	–
**Coccinellidae**									
*Adalia bipunctata* (Linnaeus, 1758)	–	–	–	1 (4)		–	–	–	–
**Cerambycidae**									
*Leptura thoracica* (Creutzer, 1799)	–	–	6 (2)	–	–	–	–	–	–
*Purpuricenus globulicollis* Dejean, 1839	–	–	–	–	1 (1)	–	–	–	0 (1)
*Purpuricenus kaehleri* (Linnaeus, 1758)	–	–	1 (1)	31 (2)	–	–	–	–	1 (4)
*Necydalis major* Linnaeus, 1758	–	–	0 (7)	32 (8)	–	–	–	0 (8)	0 (6)
*Leptura aurulenta* Fabricius, 1793	–	–	–	3 (1)	–	–	–	–	–
*Aromia moschata* (Linnaeus, 1758)	–	–	–	30 (10)	–	–	–	–	0 (8)
**Cleridae**									
*Allonyx quadrimaculatus* (Schaller, 1783)	–	–	–	2 (1)	–	0 (2)	–	–	–

* the number of sites found in the Red Data Book of the region is indicated in parentheses; ‘+’: these species are included in the Red Data Book of the Russian Federation or in the Red Data Book of the regions; ‘–‘: these species are not included Red Data Book of regions.

## Data Availability

The data is available upon request from the corresponding author.

## Appendix A

Biodiversity and occurrence of Coleoptera from fermental traps in the European part of Russia in 2018–2020.

**Table A1:** Biodiversity and occurrence of Coleoptera from fermental traps in the European part of Russia in 2018–2020.

Family, Species	2018		2019		2020	
	Number of Specimens	Occurrence, %	Number of Specimens	Occurrence, %	Number of Specimens	Occurrence, %
**Carabidae**						
*Dromius agilis* (Fabricius, 1787)					1	0.24
*Dromius quadraticollis* A. Morawitz, 1862			3	0.95		
*Harpalus distinguendus* (Duftschmid, 1812)					1	0.24
*Harpalus signaticornis* (Duftschmid, 1812)			1	0.32		
*Harpalus xanthopus winkleri* Schauberger, 1923					2	0.48
*Lebia marginata* (Geoffroy, 1785)			1	0.32		
*Limodromus assimilis* (Paykull, 1790)					2	0.48
*Limodromus krynickii* (Sperk, 1835)			1	0.32		
*Tachyta nana* (Gyllenhal, 1810)			1	0.32		
**Dytiscidae**						
*Ilybius erichsoni* (Gemminger & Harold, 1868)					1	0.24
*Ilybius fuliginosus* (Fabricius, 1792)			1	0.32		
**Hydrochidae**						
*Hydrochus brevis* (Herbst, 1793)			1	0.32		
**Histeridae**						
*Atholus duodecimstriatus* (Schrank, 1781)			1	0.32		
*Gnathoncus buyssoni* Auzat, 1917			7	1.89	6	1.19
*Platysoma elongatum* (Thunberg, 1787)			8	1.58	4	0.71
*Platysoma lineare* Erichson, 1834			7	1.89	1	0.24
**Silphidae**						
*Dendroxena quadrimaculata* (Scopoli, 1771)			3	0.95	41	5
*Necrodes littoralis* (Linnaeus, 1758)	2	2.4	21	1.58	45	4.76
*Nicrophorus humator* (Gleditsch, 1767)	1	1.2			4	0.71
*Nicrophorus interruptus* Stephens, 1830					14	1.67
*Nicrophorus sepultor* Charpentier, 1825					1	0.24
*Nicrophorus vespillo* (Linnaeus, 1758)					1	0.24
*Nicrophorus vespilloides* Herbst, 1783	20	2.4	3	0.32	4	0.71
*Oiceoptoma thoracicum* (Linnaeus, 1758)	11	9.5	24	4.1	13	2.62
*Silpha tristis* Illiger, 1798					2	0.24
**Staphylinidae**						
*Staphylinidae* sp.	13	13.1	423	22.08	252	28.57
*Philonthus* sp.	1	1.2				
*Quedius dilatatus* (Fabricius, 1787)	6	6	329	23.66	221	16.67
**Lucanidae**						
*Lucanus cervus* (Linnaeus, 1758)					4	0.71
*Platycerus caprea* (De Geer, 1774)					1	0.24
*Platycerus caraboides* (Linnaeus, 1758)			1	0.32	1	0.24
*Sinodendron cylindricum* (Linnaeus, 1758)					1	0.24
**Scarabaeidae**						
*Cetonia aurata* (Linnaeus, 1758)	60	21.4	122	17.03	635	20.48
*Esymus pusillus* (Herbst, 1789)					1	0.24
*Gnorimus variabilis* (Linnaeus, 1758)	16	7.1	33	5.99	12	2.14
*Osmoderma barnabita* Motschulsky, 1845			2	0.32		
*Protaetia affinis* (Andersch, 1797)	1	1.2			2	0.24
*Protaetia fieberi* (Kraatz, 1880)	56	33.3	250	30.28	617	29.29
*Protaetia marmorata* (Fabricus, 1792)	750	82.1	2443	67.51	2550	67.14
*Protaetia speciosissima* (Scopoli, 1786)	16	10.7	64	8.52	34	5
*Protaetia cuprea volhyniensis* (Gory & Percheron, 1833)	13	11.9	100	17.03	327	15.71
*Serica brunnea* (Linnaeus, 1758)			1	0.32	1	0.24
*Trichius fasciatus* (Linnaeus, 1758)			4	1.26		
**Scirtidae**						
*Contacyphon padi* (Linnaeus, 1758)			4	1.26		
*Contacyphon pubescens* (Fabricius, 1792)			3	0.63		
*Contacyphon* sp.			1	0.32	2	0.48
*Microcara testacea* (Linnaeus,1767)	2	1.2	1	0.32	3	0.71
**Buprestidae**						
*Agrilus sulcicollis* Lacordaire, 1835			2	0.63		
*Agrilus angustulus* (Illiger, 1803)					1	0.24
*Anthaxia quadripunctata* (Linnaeus, 1758)					1	0.24
*Buprestis haemorrhoidalis* Herbst, 1780			1	0.32		
*Dicerca alni* (Fischer von Waldheim, 1824)			1	0.32		
*Phaenops cyanea* (Fabricius, 1775)			2	0.63		
*Trachys minutus* (Linnaeus, 1758)			1	0.32		
**Eucnemidae**						
*Melasis buprestoides* (Linnaeus, 1760)					1	0.24
*Otho sphondyloides* (Germar, 1818)			1	0.32		
**Throscidae**						
*Trixagus* sp.			4	0.95	9	1.13
*Aulonothroscus* sp.			1	0.32		
**Elateridae**						
*Agriotes lineatus* (Linnaeus, 1767)					1	0.24
*Agriotes obscurus* (Linnaeus, 1758)					1	0.24
*Agrypnus murinus* (Linnaeus, 1758)			7	2.21	29	5.48
*Ampedus balteatus* (Linnaeus, 1758)			2	0.63	5	1.19
*Ampedus cinnabarinus* (Eschscholtz, 1829)			9	2.21	97	5
*Ampedus elongatulus* (Fabricius, 1787)			3	0.95	1	0.24
*Ampedus nigerrimus* (Lacordaire in Boisduval & Lacordaire, 1835)					1	0.24
*Ampedus nigrinus* (Herbst, 1784)			1	0.32		
*Ampedus nigroflavus* (Goeze, 1777)					13	1.9
*Ampedus pomonae* (Stephens, 1830)			1	0.32	13	1.67
*Ampedus pomorum* (Herbst, 1784)			4	1.26	97	9.05
*Ampedus praeustus* (Fabricius, 1792)			1	0.32	13	1.67
*Ampedus sanguinolentus* (Schrank, 1776)			3	0.95	45	4.05
*Ampedus sanguineus* (Linnaeus, 1758)					3	0.48
*Ampedus tristis* (Linnaeus, 1758)					1	0.24
*Aplotarsus incanus* (Gyllenhal, 1827)			1	0.32		
*Athous haemorrhoidalis* (Fabricius, 1801)					1	0.24
*Athous subfuscus* (O.F. Müller, 1764)			2	0.32	2	0.48
*Athous vittatus* (Fabricius, 1792)			1	0.32	4	0.95
*Cardiophorus ruficollis* (Linnaeus, 1758)			1	0.32	1	0.24
*Dalopius marginatus* (Linnaeus, 1758)	1	1.2	24	3.79	15	3.33
*Danosoma fasciatum* (Linnaeus, 1758)			1	0.32		
*Denticollis borealis* (Paykull, 1800)			1	0.32	2	0.48
*Ectinus aterrimus* (Linnaeus, 1760)			1	0.32		
*Elater ferrugineus* Linnaeus, 1758	2	2.4	6	1.89		
*Hemicrepidius niger* (Linnaeus, 1758)					1	0.24
*Lacon lepidopterus* (Panzer, 1800)					1	0.24
*Limonius minutus* (Linnaeus, 1758)			3	0.95	4	0.95
*Melanotus castanipes* (Paykull, 1800)			8	2.52	9	1.19
*Melanotus villosus* (Geoffroy, 1785)					5	0.95
*Mosotalesus nigricornis* (Panzer, 1799)			3	0.63	1	0.24
*Prosternon tesselatum* (Linnaeus, 1758)	1	1.2	14	3.47	124	9.76
*Selatosomus aeneus* (Linnaeus, 1758)			1	0.32	17	1.43
*Sericus brunneus* (Linnaeus, 1758)					1	0.24
**Lycidae**						
*Lygistopterus sanguineus* (Linnaeus, 1758)			8	1.26		
**Cantharidae**						
*Cantharis flavilabris* Fallén, 1807			1	0.32		
*Cantharis livida* Linnaeus, 1758	12	2.4	6	1.26	196	4.29
*Cantharis nigricans* O.F. Müller, 1776	2	1.2	9	2.84	90	6.9
*Cantharis pallida* Goeze, 1777					7	0.95
*Cantharis pellucida* Fabricius, 1792			10	1.26	83	5.24
*Cantharis rufa* Linnaeus, 1758			4	0.95	1	0.24
*Cantharis rustica* Fallén, 1807	1	1.2			12	0.95
*Malthodes guttifer* Kiesenwetter, 1852					2	0.48
*Malthodes* sp.					1	0.24
*Podabrus alpinus* (Paykull, 1798)					1	0.24
*Rhagonycha fulva* (Scopoli, 1763)	1	1.2			1	0.24
*Rhagonycha fugax* Mannerheim, 1843					4	0.71
*Rhagonycha lignosa* (O.F. Müller, 1764)					4	0.95
*Rhagonycha nigriventris* Motschulsky, 1860					2	0.48
**Dermestidae**						
*Attagenus schaefferi* (Herbst, 1792)			107	4.42	16	2.38
*Anthrenus museorum* (Linnaeus, 1760)			1	0.32		
*Ctesias serra* (Fabricius, 1792)			39	4.42		
*Dermestes laniarius* Illiger, 1801			1	0.32		
*Dermestes lardarius* Linnaeus, 1758			1	0.32	1	0.24
*Dermestinus murinus* Linnaeus, 1758					4	0.71
*Globicornis emarginata* (Gyllenhal, 1808)			27	2.52	17	2.14
*Megatoma undata* (Linnaeus, 1758)			1	0.32	2	0.48
*Trogoderma glabrum* (Herbst, 1783)	2	2.4	88	5.68	18	3.33
**Ptinidae**						
*Dorcatoma dresdensis* Herbst, 1792			1	0.32		
*Dorcatoma flavicornis* (Fabricius, 1792)			1	0.32		
*Dorcatoma robusta* A. Strand, 1938			6	1.58	1	0.24
**Lymexylidae**						
*Elateroides dermestoides* (Linnaeus, 1760)					1	0.24
**Cleridae**						
*Allonyx quadrimaculatus* (Schaller, 1783)			2	0.63		
*Thanasimus femoralis* (Zetterstedt, 1828)			3	0.95	2	0.48
*Thanasimus formicarius* (Linnaeus, 1758)			4	1.26	3	0.71
*Tillus elongatus* (Linnaeus, 1758)			1	0.32		
*Trichodes apiarius* (Linnaeus, 1758)	1	1.2	2	0.63	3	0.71
**Melyridae**						
*Cordylepherus viridis* (Fabricius, 1787)			1	0.32	3	0.71
*Dasytes niger* (Linnaeus, 1760)	1	1.2	10	3.15	12	1.9
*Dasytes fusculus* (Illiger, 1801)			1	0.32	7	0.95
*Malachius bipustulatus* (Linnaeus, 1758)					5	0.95
**Erotylidae**						
*Triplax russica* (Linnaeus, 1758)			2	0.63	1	0.24
*Tritoma subbasalis* (Reitter, 1896)					2	0.48
**Monotomidae**						
*Rhizophagus fenestralis* (Linnaeus, 1758)			15	2.84	39	4.29
**Nitidulidae**						
*Carpophilus hemipterus* (Linnaeus, 1758)			10	2.21	2	0.48
*Carpophilus marginellus* Motschulsky, 1858					2	0.24
*Carpophilus* sp.			1	0.32		
*Cryptarcha strigata* (Fabricius, 1787)	249	60.7	1227	44.79	1406	48.09
*Cryptarcha undata* (G.-A. Olivier, 1790)	8	3.6	14	3.78	105	8.57
*Cychramus luteus* (Fabricius, 1787)	9	6	834	12.3	101	6.9
*Cychramus variegatus* (Herbst, 1792)	4	1.2	56	5.05	15	1.43
*Cyllodes ater* (Herbst, 1792)	1	1.2			1	0.24
*Epuraea* sp.	8	8.3	268	15.77	437	19.52
*Glischrochilus grandis* (Tournier, 1872)	47	25	652	25.24	4876	50.48
*Glischrochilus hortensis* (Geoffroy, 1785)	71	25	885	28.08	783	32.38
*Glischrochilus quadriguttatus* (Fabricius, 1777)	1	1.2	33	5.68	3	0.71
*Glischrochilus quadripunctatus* (Linnaeus, 1758)	2	2.4	96	11.04	105	11.19
*Glischrochilus quadrisignatus* (Say, 1835)	2	2.4	13	2.52	75	6.19
*Meligethes* sp.			3	0.63	6	0.24
*Omosita discoidea* (Fabricius, 1775)			1	0.32		
*Pocadius ferrugineus* (Fabricius, 1775)					1	0.24
*Soronia grisea* (Linnaeus, 1758)	47	21.4	363	25.24	654	39.05
*Soronia punctatissima* (Illiger, 1794)			3	0.95	5	0.48
**Silvanidae**						
*Uleiota planatus* (Linnaeus, 1760)			1	0.32		
**Cucujidae**						
*Pediacus depressus* (Herbst, 1797)			15	3.47	29	2.86
**Laemophloeidae**						
*Cryptolestes* sp.			1	0.32		
**Cerylonidae**						
*Cerylon ferrugineum* Stephens, 1830			2	0.32		
*Cerylon histeroides* (Fabricius, 1792)			1	0.32		
**Latridiidae**						
*Corticaria* sp.					2	0.48
*Cortinicara gibbosa* (Herbst, 1793)			1	0.32	1	0.24
*Enicmus histrio* Joy & Tomlin, 1910			1	0.32		
*Stephostethus pandellei* (C.N.F. Brisout de Barneville, 1863)			2	0.63		
**Coccinellidae**						
*Adalia bipunctata* (Linnaeus, 1758)					1	0.24
*Adalia decempunctata* (Linnaeus, 1758)					1	0.24
*Anatis ocellata* (Linnaeus, 1758)			1	0.32	4	0.95
*Calvia decemguttata* (Linnaeus, 1767)	1	1.2	6	1.26	10	1.9
*Calvia quatuordecimguttata* (Linnaeus, 1758)			7	2.21	14	2.38
*Chilocorus renipustulatus* (L.G. Scriba, 1791)			2	0.63		
*Coccinella magnifica* L. Redtenbacher, 1843					2	0.48
*Coccinella septempunctata* Linnaeus, 1758					1	0.24
*Exochomus quadripustulatus* (Linnaeus, 1758)					1	0.24
*Halyzia sedecimguttata* (Linnaeus, 1758)	1	1.2	5	1.58	9	2.14
*Harmonia axyridis* (Pallas, 1773)					1	0.24
*Harmonia quadripunctata* (Pontoppidan, 1763)			2	0.63	6	1.19
*Hippodamia variegata* (Goeze, 1777)			1	0.32	1	0.24
*Mysia oblongoguttata* (Linnaeus, 1758)			1	0.32	5	1.19
*Oenopia conglobata* (Linnaeus, 1758)			1	0.32	2	0.48
*Propylea quatuordecimpunctata* (Linnaeus, 1758)					2	0.48
*Sospita vigintiguttata* (Linnaeus, 1758)			1	0.32	1	0.24
*Vibidia duodecimguttata* (Poda von Neuhaus, 1761)					1	0.24
**Mycetophagidae**						
*Litargus connexus* (Geoffroy, 1785)			17	3.47	11	1.19
*Mycetophagus quadripustulatus* (Linnaeus, 1760)			1	0.32	2	0.48
**Melandryidae**						
*Osphya bipunctata* (Fabricius, 1775)					1	0.24
*Phloiotrya subtilis* (Reitter, 1897)					1	0.24
**Mordellidae**						
*Tomoxia bucephala* A. Costa, 1854			7	1.89	1	0.24
*Mordella* sp.			3	0.63	1	0.24
**Tenebrionidae**						
*Bolitophagus reticulatus* (Linnaeus, 1767)					3	0.71
*Corticeus unicolor* Piller & Mitterpacher, 1783					2	0.48
*Lagria hirta* (Linnaeus, 1758)			19	4.73	3	0.71
*Mycetochara axillaris* (Paykull, 1799)			1	0.32		
*Mycetochara flavipes* (Fabricius, 1792)			1	0.32	1	0.24
*Upis ceramboides* (Linnaeus, 1758)			4	0.95	1	0.24
**Oedemeridae**						
*Chrysanthia geniculata* W.L.E. Schmidt, 1846					6	1.19
*Chrysanthia viridissima* (Linnaeus, 1758)	2	2.4			4	0.71
*Nacerdes carniolica* (Gistel, 1834)					38	1.67
*Oedemera femorata* (Scopoli, 1763)					1	0.24
*Oedemera virescens* (Linnaeus, 1767)					1	0.24
**Boridae**						
*Boros schneideri* (Panzer, 1796)					1	0.24
**Pyrochroidae**						
*Pyrochroa coccinea* (Linnaeus, 1760)			1	0.32	9	0.71
*Schizotus pectinicornis* (Linnaeus, 1758)			1	0.32	14	
**Salpingidae**						
*Salpingidae* sp.			1	0.32		
*Salpingus ruficollis* (Linnaeus, 1760)					1	0.24
**Aderidae**						
*Phytobaenus amabilis* R.F. Sahlberg, 1834					1	0.24
**Scraptiidae**						
*Anaspis frontalis* (Linnaeus, 1758)	1	1.2	1	0.32	5	0.95
*Anaspis thoracica* (Linnaeus, 1758)					1	0.24
**Cerambycidae**						
*Aegomorphus clavipes* (Schrank, 1781)	1	1.2	1	0.32		
*Alosterna ingrica* (Baeckmann, 1902)			1	0.32		
*Alosterna tabacicolor* (De Geer, 1775)	1	1.2	17	1.58	1	0.24
*Anaesthetis testacea* (Fabricius, 1781)			1	0.32		
*Anastrangalia reyi* (L. Heyden, 1889)			1	0.32	2	0.48
*Anoplodera rufipes ventralis* Heyden, 1886					1	0.24
*Anoplodera sexguttata* (Fabricius, 1775)	1	1.2	23	3.15	1	0.24
*Arhopalus rusticus* (Linnaeus, 1758)			5	0.95		
*Aromia moschata* (Linnaeus, 1758)	23	11.9	58	10.09	23	3.33
*Chlorophorus herbstii* (Brahm, 1790)			1	0.32		
*Cortodera femorata* (Fabricius, 1787)					4	0.71
*Dinoptera collaris* (Linnaeus, 1758)			4	1.26	5	0.95
*Etorofus pubescens* (Fabricius, 1787)			1	0.32		
*Euracmaeops marginatus* (Fabricius, 1781)			1	0.32		
*Euracmaeops septentrionis* (C.G. Thomson, 1866)			1	0.32		
*Judolia sexmaculata* (Linnaeus, 1758)			1	0.32		
*Leiopus linnei* Wallin, Nylander & Kvamme, 2009			1	0.32	2	0.48
*Leptura aurulenta* Fabricius, 1793	2	2.4	1	0.32		
*Leptura thoracica* Creutzer, 1799	68	22.6	751	31.55	1113	21.43
*Leptura quadrifasciata* Linnaeus, 1758	104	35.7	489	37.22	433	19.29
*Lepturalia nigripes* (De Geer, 1775)	2	1.2	14	1.89	57	4.76
*Lepturobosca virens* (Linnaeus, 1758)					5	0.48
*Mesosa myops* (Dalman, 1817)	5	3.6	13	2.84	1	0.24
*Molorchus minor* (Linnaeus, 1758)			3	0.95	12	2.38
*Monochamus sutor* (Linnaeus, 1758)			1	0.32		
*Necydalis major* Linnaeus, 1758	9	7.1	47	10.73	32	5.71
*Nivellia sanguinosa* (Gyllenhal, 1827)			1	0.32		
*Obrium cantharinum* (Linnaeus, 1767)	1	1.2	45	5.68	104	7.14
*Oedecnema gebleri* (Ganglbauer, 1889)					2	0.48
*Pachyta quadrimaculata* (Linnaeus, 1758)	4	3.6	5	1.58	3	0.71
*Phymatodes testaceus* (Linnaeus, 1758)			2	0.32	24	2.86
*Plagionotus arcuatus* (Linnaeus, 1758)					2	0.48
*Plagionotus detritus* (Linnaeus, 1758)	1	1.2	8	2.21	119	2.38
*Prionus coriarius* (Linnaeus, 1758)			1	0.32		
*Purpuricenus globulicollis* Dejean, 1839			5	0.95	1	0.24
*Purpuricenus kaehleri* (Linnaeus, 1758)	7	7.1	124	11.04	167	7.62
*Rhagium inquisitor* (Linnaeus, 1758)			24	4.73	10	1.9
*Rhagium mordax* (De Geer, 1775)	49	20.2	422	24.29	778	34.05
*Rhagium sycophanta* (Schrank, 1781)	2	2.4				
*Rhamnusium bicolor* (Schrank, 1781)	1	1.2				
*Ropalopus clavipes* (Fabricius, 1775)					1	0.24
*Ropalopus macropus* (Germar, 1823)					2	0.24
*Rutpela maculata* (Poda von Neuhaus, 1761)	9	7.1	20	3.15	16	2.38
*Saperda scalaris* (Linnaeus, 1758)					1	0.24
*Spondylis buprestoides* (Linnaeus, 1758)			2	0.63	1	0.24
*Stenocorus meridianus* (Linnaeus, 1758)	7	8.3	93	11.67	38	1.9
*Stenurella melanura* (Linnaeus, 1758)					1	0.24
*Stictoleptura maculicornis* (De Geer, 1775)			2	0.63	2	0.48
*Stictoleptura rubra* (Linnaeus, 1758)			2	0.63	1	0.24
*Stictoleptura variicornis* (Dalman, 1817)			1	0.32		
*Strangalia attenuata* (Linnaeus, 1758)			8	1.89	2	0.48
*Trichoferus campestris* (Faldermann, 1835)	2	2.4	7	0.95	1	0.24
*Xylotrechus antilope* (Schoenherr, 1817)			17	3.47	34	4.29
*Xylotrechus arvicola* (Olivier, 1795)	1	1.2			2	0.48
*Xylotrechus capricornus* (Gebler, 1830)					1	0.24
*Xylotrechus pantherinus* (Savenius, 1825)			2	0.63	1	0.24
*Xylotrechus rusticus* (Linnaeus, 1758)			3	0.95	1	0.24
**Chrysomelidae**						
*Altica* sp.			5	1.58	8	1.43
*Aphthona* sp.					1	0.24
*Chrysomela vigintipunctata* (Scopoli, 1763)					1	0.24
*Crepidodera aurata* (Marsham, 1802)			1	0.32		
*Crepidodera nitidula* (Linnaeus, 1758)			1	0.32		
*Galerucella lineola* (Fabricius, 1781)			2	0.63	2	0.48
*Gonioctena viminalis* (Linnaeus, 1758)					1	0.24
*Hypocassida subferruginea* (Schrank, 1776)					1	0.24
*Lochmaea caprea* (Linnaeus, 1758)			2	0.32		
*Orsodacne cerasi* (Linnaeus, 1758)					2	0.48
*Phyllotreta undulata* Kutschera, 1860					1	0.24
*Plagiosterna aenea* (Linnaeus, 1758)			1	0.32	1	0.24
**Anthribidae**						
*Dissoleucas niveirostris* (Fabricius, 1798)					1	0.24
*Tropideres albirostris* (Schaller, 1783)			2	0.63	5	1.19
**Attelabidae**						
*Byctiscus betulae* (Linnaeus, 1758)					1	0.24
**Brentidae**						
*Betulapion simile* (Kirby, 1811)			1	0.32	1	0.24
**Curculionidae**						
*Anisandrus dispar* (Fabricius, 1792)	1	1.2	386	10.73	2012	17.86
*Anthonomus incurvus* (Panzer, 1795)			1	0.32		
*Bagous puncticollis* Boheman, 1845			1	0.32		
*Brachyderes incanus* (Linnaeus, 1758)			2	0.63		
*Coeliodinus rubicundus* (Herbst, 1795)			2	0.63		
*Curculio glandium* Marsham, 1802			1	0.32	1	0.24
*Curculio nucum* Linnaeus, 1758	2	2.4	1	0.32	16	0.48
*Curculio venosus* (Gravenhorst, 1807)			1	0.32		
*Curculio villosus* Fabricius, 1781					4	0.95
*Ellescus bipunctatus* (Linnaeus, 1758)			1	0.32		
*Ellescus scanicus* (Paykull, 1792)			1	0.32		
*Hylastes opacus* Erichson, 1836			2	0.63		
*Ips acuminatus* (Gyllenhal, 1827)			1	0.32		
*Ips typographus* (Linnaeus, 1758)					1	0.24
*Orchestes rusci* (Herbst, 1795)			1	0.32	1	0.24
*Phyllobius arborator* (Herbst, 1797)			1	0.32		
*Phyllobius argentatus* (Linnaeus, 1758)			6	1.26	5	1.19
*Phyllobius maculicornis* Germar, 1823			2	0.63	1	0.24
*Phyllobius pomaceus* Gyllenhal, 1834			1	0.32		
*Phyllobius pyri* (Linnaeus, 1758)			4	0.63	6	1.43
*Pissodes piniphilus* (Herbst, 1797)					1	0.24
*Polydrusus cervinus* (Linnaeus, 1758)			1	0.32		
*Polydrusus flavipes* (De Geer, 1775)	1	1.2	1	0.32		
*Polydrusus* sp.					1	0.24
*Polydrusus tereticollis* (De Geer, 1775)					1	0.24
*Polygraphus subopacus* C.G. Thomson, 1871			1	0.32		
*Scolytus intricatus* (Ratzeburg, 1837)					1	0.24
*Sitona ambiguus* Gyllenhal, 1834					1	0.24
*Sitona macularius* (Marsham, 1802)					1	0.24
*Strophosoma capitatum* (De Geer, 1775)			5	1.26	7	1.43
*Trypodendron signatum* (Fabricius, 1792)			1	0.32		
*Xyleborus saxesenii* (Ratzeburg, 1837)			92	1.26	123	3.09
TOTAL	1750		11,655		19,864	
